# Impact of Storing Condition on Staling and Microbial Spoilage Behavior of Bread and Their Contribution to Prevent Food Waste

**DOI:** 10.3390/foods10010076

**Published:** 2021-01-02

**Authors:** Thekla Alpers, Roland Kerpes, Mariana Frioli, Arndt Nobis, Ka Ian Hoi, Axel Bach, Mario Jekle, Thomas Becker

**Affiliations:** 1 Research Group Cereal Technology and Process Engineering, Brewing and Beverage Technology, Technical University of Munich, 85354 Freising, Germany; thekla.alpers@tum.de (T.A.); mjekle@tum.de (M.J.); tb@tum.de (T.B.); 2 Research Group Beverage Biotechnology, Brewing and Beverage Technology, Technical University of Munich, 85354 Freising, Germany; frioli.maa@gmail.com; 3 Research Group Raw Material Based Brewing and Beverage Technology, Brewing and Beverage Technology, Technical University of Munich, 85354 Freising, Germany; arndt.nobis@tum.de (A.N.); kaian.hoi@tum.de (K.I.H.); 4 Science Journalist, 50670 Cologne, Germany; axelbach@gmx.de

**Keywords:** shelf life, household storage methods, firming, texture analysis, mycotoxins, sourdough, wheat pan bread, mixed-type sourdough bread, food waste

## Abstract

The high loss rate of bread is generally known to contribute to the alarmingly high numbers in worldwide food waste. Correct storage techniques are believed to enable the reduction of preventable food waste. Therefore, the influence of storage parameters on staling and spoilage behavior of German bread within the limits of common household methods was investigated in this study. The aim was to generate reliable data for staling and spoilage using different storage methods (PE-layered microperforated paper bag, plastic bag, and fridge and bread box) to bridge the gap between consumer’s needs and scientific research questions. Everyday routines of life, such as visual inspection, were compared with microbiological techniques and were found to represent an adequate tool for microbial safety control. Visually undetectable fungal growth has not been found to result in the production of mycotoxins (fumonisins B1 and B2 and ochratoxin A) in quantifiable or harmful concentrations. Thus, disgust should prevent any foodborne health risks as the visual appearance should lead to avoiding the consumption of spoiled food before mycotoxins are produced in amounts causing adverse health effects within the limits of this experimental setup. Additionally, the storage temperature especially was found to influence the kinetics of staling processes, as a reduction accelerated the staling process. Further, crumb moisture loss was found to contradict a long shelf life but, on the other hand, an elevated humidity was shown to provoke excessive microbial growth and should therefore be observed when designing suitable storage methods. Further, the correct choice of the bread type stored and a good sanitary practice represent simply accessible ways to prolong the storage period of bread loaves.

## 1. Introduction

Staling and microbial spoilage of bread are the two main reasons for the alarmingly high contribution of bakery products to food waste all over the world (34.7% of the total amount of baked goods in Germany in 2015) [1]. Within the storage period of bread, several important changes contribute to the decreasing consumer acceptance of stored bread. These changes include physicochemical processes such as the crumb firming process, water migration within the crumb, crust and the environment, the loss of flavor and microbial spoilage. Even though there has been respectable research addressed to this topic over the years, there is a lack of published research on the optimal consumer storage conditions for bakery products to minimize waste.

In general, the staling process describes the mechanism of bread aging and is initiated immediately after baking. With the termination of the input of thermal energy, phase transition processes occur and modify the texture of bread. During the first hours after baking, the recrystallization of amylose positively impacts the solidification of the crumb structure, whereas amylopectin, the second main macromolecule accounting for the starch fraction in flour, crystalizes over a longer period of days [2,3,4]. This retrogradation process is also responsible for the redistribution of water on the molecular level as the recrystallization of starch is accompanied by the increased formation of B-type crystalline polymorphs, which are capable of immobilizing more water molecules than A-type crystals [5]. Thus, water is removed from the gluten network, leading to a less elastic and firmer texture [6]. Further water migration processes occur on a macrostructural level as water is exchanged within the moisture crumb and the dry crust according to the differences in vapor pressures between crumb and crust until the mass equilibrium is reached [7]. The long-term processes of starch retrogradation and water migration are further accompanied by the evaporation of volatile components, leading to a change in consumer perception even within the first days of storage [8]. The physicochemical changes taking place during the storage period of bread can be traced by textural analysis. Transformations can be quantified by human and instrumental analysis, including sensory evaluations, uniaxial compression testing (according to AACCi method 74-10A) or dynamic mechanical analysis.

Beside textural transformations, consumer acceptance is affected by microbial spoilage. As bread represents an excellent source for microbiological growth, it is likely to be subjected to fungal contamination, limiting the shelf life. The intrinsic composition of bread (≈40%(*w*/*w*) water content, pH = 5.5–6.0 and an a_w_-value within 0.94–0.97) facilitates fungal growth [9]. As spores are commonly considered to be inactivated during the baking process [10], contamination generally arises from the surrounding air, machines, workers (production and sale), the consumer or the storage environment [9,11,12]. Such contaminations lead to fungal growth, which can result in undesired changes causing consumer rejection due to the development of mycelium or the accumulation of mycotoxins in the bread matrix, which can cause adverse health outcomes involving carcinogenic or genetically harmful effects [13]. Commonly detected fungi, molds and bacteria include *Penicillium* spp., *Aspergillus* spp., *Bacillus* spp. and *Cladosporium* sp., with spoilage having been shown to be dominated by *Aspergillus* spp. in warmer countries, whereas *Penicillium* spp. predominate under moderate climate conditions [9]. A review of common bread spoilage fungi is available [13]. In order to avoid preventable food waste, several preservation methods are commonly involved in production, handling and storage processes. Beside adequate and sanitary production conditions, the usage of sourdough has been reported to reduce the growth rate of fungi, thus prolonging the shelf life of bakery products [14,15,16,17]. Such procedures could help reducing the alarmingly large amount of worldwide food waste, together accounting for one third of the food produced for human consumption. Bread and cereal products contribute to food waste with a loss rate of 26.3% during production, retail and wholesale and consumption in Germany [18]. Further, storage conditions can significantly affect the staling and spoiling behavior of bread. Parameters such as storage temperature and humidity are well-known to influence the retrogradation process and growth rate of microorganisms [19,20,21].

The aim of this study is therefore to evaluate the impact of different storage parameters on the shelf life of bread. It is hypothesized that a targeted selection of storage parameters within the limits of ordinary household storage methods can attribute to a prolonged shelf life for commonly consumed German bakery products, such as pan wheat bread and mixed-type sourdough bread. Therefore, the impact of four different storage methods was compared in terms of firming rate, changes in the visual appearance, the concentration of fungi measured by plate counting and the accumulation of mycotoxins during a storage period of ten days. This could help solving uncertainties among consumers regarding microbial safety and reduction of food waste due to undesirable textural appearance.

## 2. Materials and Methods

### 2.1. Bread Storage and Sampling

**Figure A1:**
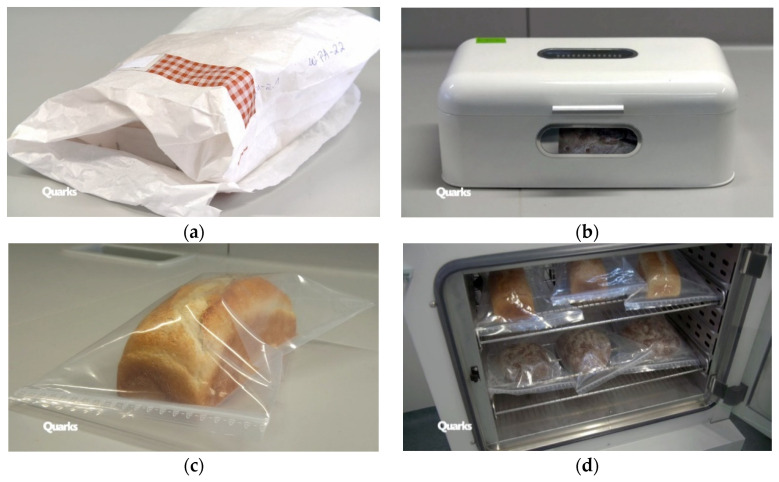
Exemplary presentation of the storage setup. The bread loaves were stored in four different common storage methods. (**a**) Bread loaf stored in a PE-layered microperforated paper bag (21.3 ± 0.4 °C). (**b**) Mixed-type sourdough bread stored in a bread box with 13 little ventilation holes on the back side (22.0 ± 0.5 °C). (**c**) Pan wheat bread stored in a plastic bag at room temperature (21.9 ± 0.5 °C). (**d**) Pan wheat bread and mixed-type sourdough bread stored in plastic bags at fridge temperature (8.2 ± 0.2 °C). © WDR (Westdeutscher Rundfunk).

In order to represent everyday life circumstances, pan wheat bread and mixed-type sourdough bread were purchased at a local bakery store in Freising, Germany. All samples originated from one manufacturing batch. According to the supplier, the wheat bread contained wheat flour, water, sodium chloride and yeast, whereas the mixed-type sourdough bread contained rye and wheat flour (70:30, *w*/*w*), water, sourdough, sodium chloride and *S. cerevisiae* sp. After purchasing, four different common storage methods were compared: (1) metal bread box (BB) at 22.0 ± 0.5 °C, (2) polyethylene (PE)-layered microperforated paper bag with fine ventilation holes every 1.5 cm (purchased at a local bakery) at 21.3 ± 0.4 °C (LP), (3) low-density polyethylene (LDPE)-plastic bag (250 × 350 × 0.09 mm, dm-folien GmbH, Reutlingen, Germany) at 21.9 ± 0.5 °C (PR) and (4) LDPE-plastic bag at 8.2 ± 0.2 °C, representing storage in a fridge (PF). The temperature and moisture for the storage method applied were permanently logged using a temperature and humidity logger (OM-EL-USB-2-LCD, Omega, Deckenpfronn, Germany). An exemplary storage setup can be seen in Figure A1. In order to provide enough sample material, two bread loaves were stored together in one container for the pan wheat bread, whereas one loaf of bread was stored per container for the mixed-type sourdough bread. For a period of ten days, bread slices of 1.25 ± 0.25 cm were obtained daily from the open end of the breads, imitating typical consumer behavior. The sampling and handling of slices was performed with great care, using gloves and deploying surface disinfection methods after taking each sample, to avoid any contamination from external sources and cross-contamination.

### 2.2. Initial Bread Characterization

An initial characterization of the purchased breads was conducted on day 0 of the storage period. The pH value and total titratable acidity (TTA) were analyzed according to the guidelines of the International Association for Cereal Science and Technology (ICC) [22], using a pH meter (206-pH2, Testo SE & Co. KGaA, Lenzkirch, Germany) and a Titrator (Titroline 6000, SI Analytics GmbH, Mainz, Germany) equipped with 0.1 M NaOH (Merck KGaA, Darmstadt, Germany). Water activity (a_w_-value) was measured with an electronic hygrometer (Aqua Lab, Decagon Devices, Pulmann, WA, USA). Using the chilled-mirror dew-point technique, the a_w_-value was determined and previously calibrated with standard solutions of NaCl (6 M, VWR International GmbH, Darmstadt, Germany) and KCl (0.5 M, Carl Roth GmbH & Co. KG, Karlsruhe, Germany) of known activity. All measurements were performed at a sample temperature of 25 °C.

### 2.3. Moisture Determination

The moisture content of the sampled slices was determined using an oven drying method at 130 °C for 16 h. The moisture content was calculated as the weight difference in relation to the original sample weight. For standardization of the sample preparation, the samples were stored in sealed plastic bags overnight at −33 °C before moisture measurements were conducted.

### 2.4. Texture Profile Analyses

Crumb firming behavior was accessed using instrumental texture profile analysis. Two bread slices (wheat bread: first slice of two breads which were stored under the same conditions; mixed-type sourdough bread: halves of the first slice stacked upon each other), each with a thickness of 12.5 mm, were analyzed using a Texture Analyser (TVT-300 XP, 5 kg load cell, Perten Instruments, Hägersted, Schweden). The firmness of the samples was measured in two compression cycles to up to 40% of the original height. The following test settings were applied: a test speed of 1.7 mm/s, a post-test speed of 1.0 mm/s, a trigger force of 0.05 N and a relaxation time of 5 s between the measurement cycles. The measurements were performed after sufficient resting time in order to compensate potential temperature differences.

### 2.5. Plating and Identification of Spoilage Organisms

The count of viable and cultivable yeast was evaluated following the ICC standard method No. 146 for yeasts. The sampled slices were prepared for microbiological analysis by grinding with liquid nitrogen to allow for a homogeneous and complete extraction of yeasts and molds during the consecutive extraction process. The extraction was performed with Ringer’s solution (VWR International GmbH, Darmstadt, Germany). Suitable dilutions of 1 mL (10^−2^ and 10^−4^) were spread on malt extract agar and incubated at 28 °C for 2 days. The progress of microorganism growth was documented and the individual colonies were identified microscopically. Isolation of individual colonies was performed for further identification.

### 2.6. Mycotoxins

#### 2.6.1. Fumonisins B1 and B2

Analysis for the fumonisins B1 and B2 was performed by liquid chromatography with tandem mass spectrometry (LC-MS/MS) according to the method developed by [23]. First, 0.5 g of the grinded samples was extracted using 2 mL of extraction solution (acetonitrile/water/acetic acid 79:20:1, *v*/*v*/*v*). After 90 min of extraction using a REAX2 shaker (Heidolph Instruments GmbH & CO. KG, Schwabach, Germany), the solid parts were removed by centrifugation (2 min at 3000 rpm, Eppendorf AG, Hamburg, Germany) and the extracts were diluted 1:1 (*v*/*v*) with a dilution solvent (acetonitrile/water/acetic acid 20:79:1, *v*/*v*/*v*). An injection volume of 5 µL was filtered using a 0.45 µm membrane and injected into the LC-MS/MS system operating at a flow rate of 1 mL/min. The system used for detection and quantification included a 1200 series HPLC-System (Agilent, Waldbronn, Germany) consisting of a HiP-ALS SL autosampler, a 1200 series bin pump module, a 1200 series degasser and a 1100 series column oven, coupled to the Triple Quad 4500 MS (SCIEX, Darmstadt, Germany). The ion spray voltage was set to 4.000 V, the curtain gas pressure was set to 10 psi, the nebulizer gas pressure was 50 psi and the heater gas pressure was 50 psi. The turbogas temperature was set to 550 °C. The measurement was performed in the multiple reaction monitoring (MRM) mode in positive mode. Further, the system was equipped with a Gemini^®^ C18 column, 150 × 4.6-mm i.d., 5-μm particle size column (Phenomenex, Torrance, CA, USA). Eluent A was a 5 mM ammonium acetate, composed of methanol/water/acetic acid 10:89:1, *v*/*v*/*v*) and eluent B was a 5 mM ammonium acetate, composed of methanol/water/acetic acid 97:2:1, *v*/*v*/*v*). The bivariate gradient was set as follows: 2 min at 100% eluent A followed by a linear increase of eluent B to 100% over 12 min, followed by an isocratic phase of 3 min at 100% eluent B and finally a 4 min re-equilibration phase at 100% eluent A. Isotope-labeled internal standards of fumonisins B1 and B2 were used for the quantification. The limits of detection were 11.2 µg/kg and 8.2 µg/kg for fumonisins B1 and B2 respectively. The limits of quantification were 30.3 µg/kg and 22.7 µg/kg, following the abovementioned order.

#### 2.6.2. Ochratoxin A (ELISA)

For determination of ochratoxin A, RIDASCREEN^®^ Ochratoxin A 30/15 (R-Biopharm AG, Darmstadt, Germany) was conducted as a competitive ELISA. The method was carried out according to the manufacturer’s guidelines [24].

### 2.7. Statistical Analysis

All measurements were performed in triplicates. The stated standard deviation accounts for the deviation between triplicates. Statistical evaluation was performed using Origin (2018b, OriginLab Corporation, Northampton, MA, USA).

## 3. Results and Discussion

### 3.1. Initial Bread Characteristics

Since the aim of this study was to elaborate the spoilage and staling behavior of bread under everyday life circumstances, the bread analyzed in this study was purchased in a local bakery. An initial characterization was performed to access the main parameters known to impact the storage behavior of bread. For the analyses, a representative initial sample was taken of each bread type on day 0. The results of the initial specification are presented in Table 1 in terms of moisture content, water activity (a_w_-value) and acidity (pH-value and TTA). These intrinsic factors are well known to be decisive for microbial growth. The aim of the initial characterization was to predict the likelihood of spoilage and staling for both bread types. It appears that the moisture content of the mixed-type sourdough bread (48.59% ± 0.01%) was slightly higher compared to the water content of the pan wheat bread (45.38% ± 2.60%). These differences were likely caused by the different flour types and recipes used for the bread production. In general, the higher content of non-starch polysaccharides (pentosans, β-glucan, lignin and cellulose, arabinoxylan) in rye flour leads to higher water-absorbing properties [25] and thus to a higher water addition. Further, a better gelling potential has been reported for rye-originated arabinoxylans compared to wheat endosperm-originated arabinoxylans due to structural differences [26,27]. Therefore, the higher moisture content of the mixed-type sourdough bread in this experiment was likely to be caused by the higher water addition. Regarding the influence of the different moisture contents on the storage behavior, higher water content is known to affect the staling process but does not determine the spoilage process. For the latter, water activity is the decisive factor. The water activity is defined as the ratio of the vapor pressure of water over a substrate related to that over pure water at the same temperature and pressure. A high a_w_-value can promote microbial growth, whereas high osmotic pressure can result in dehydration, leading to a lack of growth ability and potential cell death. Thus, water activity is a limiting factor for food spoilage. Having a rather high water activity, bread is subjected to all kinds of spoilage since the lower limits for bacteria, yeast and molds are comparatively low. The a_w_-values of both bread types are presented in Table 1 and suggest that both bread types would be highly exposed to food spoilage [9]. According to hurdle technology, which is commonly used to evaluate the microbial safety of food and is based on the combination of several preservation factors, the acidity can be used to prolong the shelf life of food products and counteract a high spoilage possibility due to high moisture content [28]. From Table 1 it can be seen that the pH value of the mixed-type sourdough bread was lower compared to the pan wheat bread and the total titratable acidity was comparably high. Both values were within the advised range of typical values for the respective product type [29]. In general, the higher acidity of the mixed-type sourdough bread has its origin in the incorporation of sourdough. During the sourdough fermentation step acids are produced which contribute to the taste and a longer shelf life [17,30]. The use of sourdough is necessary to produce rye bread, as this is a common way to reduce the enzymatic activity of α-amylase [31]. The higher acidity of the mixed-type sourdough bread might have contributed to a lower spoilage probability and a better microbial stability in the storage trials performed within this study.

### 3.2. Microbial Stability of Breads under Different Storage Conditions

To access the microbial stability of bread under different storage conditions, two different methods were applied in this study. Visual inspection of the bread loaves was performed and validated using microbiological techniques. In this setup, the visual appearance was meant to represent the consumer’s test possibilities and the results were related to the results of plate counting experiments as a common reference technique for the identification of microbial spoilage. By comparing the spoiling results of the consumer’s technique and those of the laboratory approach, the question of whether visual appearance represents a suitable tool for the consumer to judge microbial safety was able to be answered.

**Figure A2:**
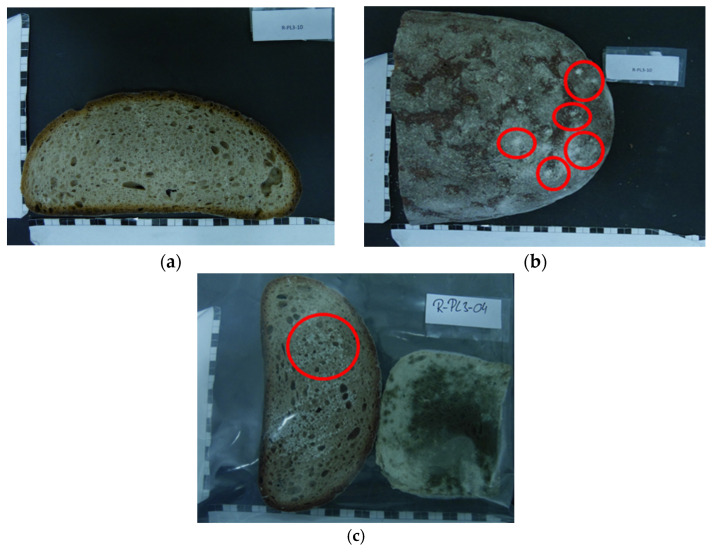
Growth of molds on mixed-type sourdough bread stored in a plastic bag at room temperature. (**a**) Crumb after ten days of storage without visually detectable growth of molds. (**b**) Top view of the bread loaf showing the first molds after ten days of storage. (**c**) Cross-contaminated mixed-type sourdough bread slice after four days of storage with a spoiled pan wheat bread slice, highlighting the importance of sanitary storage conditions.

**Figure A3:**
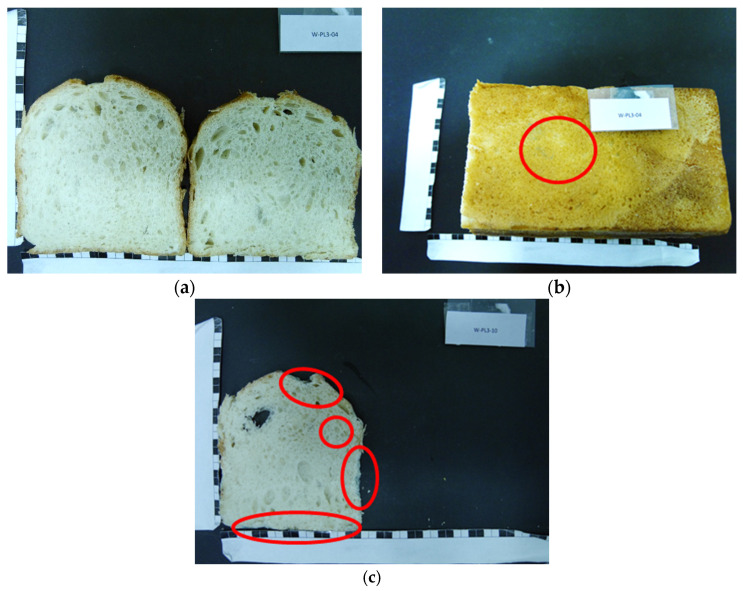
Growth of molds on pan wheat bread stored in a plastic bag at room temperature. (**a**) Crumb after four days of storage without visually detectable growth of molds. (**b**) Bottom view of the bread loaf showing the first molds after four days of storage. (**c**) Crumb with excessive growth of molds at the side, top and bottom after ten days of storage.

Initial plate counting experiments confirmed the microbial safety at the beginning of the storage period. As expected, no yeasts were present on day 0. During the whole storage period of ten days, the mixed-type sourdough bread was shown to be more stable compared to the pan wheat bread. Spoilage of mixed-type sourdough bread occurred significantly later than for wheat pan bread and in only one of the four different storage methods. For mixed-type sourdough bread, no growth of molds was visually detectable until day 10 for any kind of storage method (see Figure A2). Only the storage in plastic bags resulted in spoilage after day 10 (*n* = 1) or 13 (*n* = 3). Using microbiological analysis, the spoiling organisms were identified as *Penicillium* spp. As the main purpose of this study was to test different ordinary household storage methods for their suitability for prolonging the shelf life of bread, the following comparison focuses on the spoilage behavior of pan wheat bread. For this bread type, substantial differences were observed between the different storage methods. Storage in a plastic bag at room temperature was the first storage method subjected to visually detectable mold growth (see Figure A3). The visual evaluation revealed the growth of molds from day 4 (*n* = 1) and 5 (*n* = 3) on. The molds were found to grow on the wheat bread crust, while the crumb was without any mycelium until day 10. This is believed to be caused by the experimental setup, as the slice subjected to the surrounding atmosphere was renewed after every 24 h. The plate counting revealed the presence of molds even before their visual appearance. *Penicillium* spp. showed exponential growth after a long lag phase from day 4 (5 colony forming units (CFU)) on and *Cladosporium* spp. was detected at day 8. In general, *Penicillium* spp. are known to commonly dominate the microbial spoilage on bread under moderate climate conditions [9]. The excessively humid atmosphere (89.9% rh) dramatically provoked the growth of molds. Even the sourdough containing mixed-type bread was subjected to *Penicillium* spp. after 10 days of storage in a plastic bag at room temperature. No other storage method caused the spoilage of mixed-type sourdough bread.

The storage in bread boxes caused a longer lag phase and slower growth rates. The first visual appearance of mycelium appeared at day 4 (*n* = 1), 5 (*n* = 2) or 6 (*n* = 3). Bread boxes might commonly be expected to represent the most stable storage method, but within this experiment they were shown to provoke a highly humid atmosphere (89.0% rh). Even waterlogging was found to occur due to the limited air circulation underneath the bread loaves, promoting the growth of molds at this position. Consequently, molds were primarily found on the bottom of the breads, where moldy build-up of damp was found to be strongest. Using plate counting experiments, *Aspergillus* spp. were detected for the first time at day 2 (3 CFU) and presented exponential growth until day 10. As *Aspergillus* spp. are unlikely to dominate the spoilage under moderate conditions, but are ubiquitously present in the form of endospores, it is likely that the *Aspergillus* spp. infection occurred as a secondary infection. This organism would probably have infected the plastic bag-stored wheat bread as well, but the *Penicillium* spp. dominated the mixed culture there. Beside *Aspergillus* spp., *Penicillium* spp. were identified on the wheat pan bread stored in the bread boxes and were found to grow exponentially from day 6 on. Further, *Cladosporium* spp. were detected at day 8.

For paper bag storage, the formation of a mycelium was detectable from day 5 (*n* = 2) or 6 (*n* = 3) on. In comparison with the other storage methods, the humidity inside the paper bag was found to be significantly lower due to the higher water permeability of the PE-layered microperforated paper (80.5% rh). This was found to reduce the growth rate of *Penicillium* spp., leading to the first appearance of *Penicillium* spp. for the plating experiments on day 6. *Aspergillus* spp. were detected for the first time on day 1 (*n* = 1, 1 CFU) or 2 (*n* = 3, 2 CFU) and presented exponential growth until day 10, probably due to the same reasons as for the appearance of *Aspergillus* spp. in the bread box.

It is generally known that the growth of molds is preventable with storage at refrigerator temperature. Therefore, the storage of bread in plastic bags under refrigerator conditions was included in the comparison of storage methods as well. Even though the plastic bag was shown to promote the strongest spoilage at room temperatures, no mold growth was detected upon storage in the same wrapping at 8 °C for a storage period of 10 days by visual inspection nor plate counting. In this case, temperature was shown to be the limiting factor, as the measured humidity was comparable to the humidity measured in the plastic bags stored at room temperature.

Further experiments were performed to evaluate the effect of sanitary conditions on bread spoilage. A moldy slice of wheat bread and a non-moldy slice of mixed-type sourdough bread were placed together in a plastic bag and stored at room temperature. The moldy wheat pan bread slice was used to simulate a high mold load in the storage container as would be the case for an insanitary storage environment. The successful cross-contamination of mixed-type sourdough bread after 4 (*n* = 1) to 5 days (*n* = 3) proved the strong impact of the hygienic conditions during bread storage (see Figure A2).

From the results it is apparent that humidity and temperature varying in the range of typical household storage methods are the main factors influencing the microbial shelf life of bread. Reducing the temperature to refrigerated temperatures was shown to be able to decelerate the growth rate of fungi. Further, the occurrence of high humidity due to inappropriate wrapping materials or container design was found to promote microbial spoilage. Consequently, both factors should be considered when designing suitable storage methods for bread. Further, a good sanitizing practice was shown to be able to prolong the shelf life of bread, as a high load of molds was shown to reduce the storage time significantly. Visual testing was found to be less sensitive for the detection of microbial growth. In general, the presence of molds and yeasts causes disgust, though they do not represent harm for human health in general. Nevertheless, food safety is affected by mycotoxins, the production rate of which is not linked to the same parameters as fungal growth. Therefore, food safety can only be judged by testing for the occurrence of mycotoxins.

### 3.3. Relation between Microbial Growth and Mycotoxin Production

The most relevant mycotoxins in food are aflatoxins, ochratoxins, patulin, fumonisins, zearalenone and trichotecenes [32,33]. In the present study, ochratoxin A (OTA) and fumonisins B1 and B2 were quantified. These mycotoxins are commonly produced by several *Penicillium* and *Aspergillus* spp. and *Fusarium* and *Aspergillus* spp., respectively. Both are considered as potentially carcinogenic, immune-suppressive and nephrotoxic. Fumonisins are further regarded as hepatotoxic [33]. The maximum permitted level of daily OTA intake is 3 ng/g in consumed cereal products derived from unprocessed cereals according to the EU commission regulation (EC) No 1881/2006. The mold colonies detected in the storage trials were found to produce mycotoxins, as OTA and fumonisins B1 and B2 were detected in isolated colonies. Nevertheless, the quantification of OTA in bread slices was not successful as the amount of the mycotoxin was below the limit of detection. Consequently, the level of OTA in bread slices sampled on day 10 was lower than the limit of detection of the applied method (1.25 µg/kg). The daily intake of ochratoxin A from cereal-based products should not exceed 3 µg/kg according to the EU commission regulation No 1881/2006. Regarding fumonisins B1 and B2, the upper limit suggested for the daily intake is 1000 µg/kg in maize-based foods. Using the LC-MS method described above, no fumonisin B1 or B2 could be quantified in bread slices after 10 days of storage in plastic bags at room temperature, even though this storage method resulted in excessive spoilage. According to this finding, the levels of fumonisins B1 and B2 were below the suggested upper limit of daily intake, as the limits of quantification were 30.3 µg/kg and 22.7 µg/kg for fumonisins B1 and B2, respectively. It can therefore be concluded that all breads were microbially safe and did not represent any health risk.

### 3.4. Bread Staling under Different Storage Conditions

Next to microbial spoilage, staling is the major reason for decreasing consumer acceptance with increasing storage time. Bread staling involves several different processes, such as water redistribution and phase transitions. During baking, water is redistributed among starch and gluten according to their gelling capacity in the crumb and is evaporated from the crust. After the baking process, water is redistributed on a molecular and macroscopic scale: water is exchanged between the dry crust and the moisture crumb (crust staling) and within the crumb, where water is transferred from gluten to starch due to recrystallization (crumb staling). In a closed system, an equilibrium would be reached after sufficient time and a constant moisture content of crumb and crust would be set. If the crust can no longer serve as barrier against moisture loss from the crumb, as would be the case after cutting the bread, the bread is subjected to dehydration.

The moisture of the stored breads showed a stepwise decrease from day 0 to day 1 as the crust, serving as an intrinsic barrier, was removed and the first slice was subjected to dehydration. The different storage methods were shown to result in different absolute moisture losses, as presented in Table 2. Drying out was best prevented by the storage in a plastic bag. The plastic served as an artificial barrier and hindered the air circulation with the surrounding atmosphere. The chosen plastic wrapping had a low permeability for water and caused the establishment of an equilibrium between crumb, crust and air within the plastic bag. The overall decrease of moisture can be attributed to the daily sampling procedure and the small amount of water that was absorbed from the gas within the packaging. The storage temperature did not affect the moisture loss or the humidity in plastic bags. The humidity inside plastic bags was 89.9% and 91.3% for room temperature and fridge temperature, respectively. Storage inside a bread box showed the second highest moisture loss throughout the storage time. The bread box reduced the free exchange of air but not to the same extend as a sealed plastic bag. This was further confirmed by the humidity inside this storage container, which was traced during the storage of the breads and was found to be rather high in bread boxes (89.0%). The rather high humidity can be attributed to limited air circulation and provoked the slow moisture loss of the stored breads. The storage in a PE-layered microperforated paper bag at room temperature resulted in the highest moisture loss. The PE-layered microperforated paper had the highest water permeability and therefore offered the lowest barrier properties. Consequently, the humidity inside the paper bag was the lowest found among the different storage conditions and the closest to the humidity of the surrounding laboratory (rh = 40.5%). On the whole, the absolute moisture loss was higher for mixed-type sourdough breads for all storing methods. The faster drying might be attributed to the higher initial moisture content of this bread type. As the differences in vapor pressures between the surrounding atmosphere and the crumb were higher, a higher water transfer rate might have occurred.

**Figure 1:**
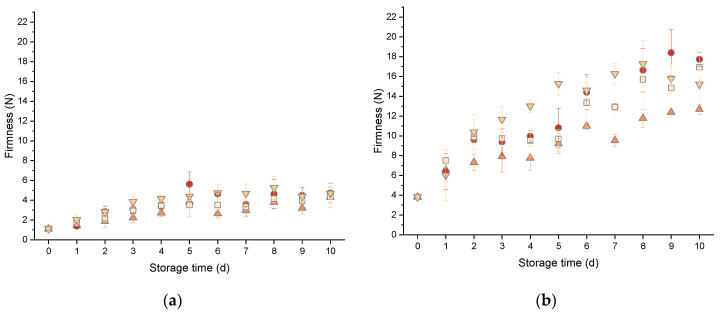
Bread firmness as a function of the storage time depending on the storage conditions applied. (**a**) Pan wheat bread. (**b**) Mixed-type sourdough bread. (■) Bread box at room temperature (BB), (●) PE-layered micro perforated paper bag at room temperature (LP), (▲) plastic bag at room temperature (PR) and (▼) plastic bag fridge temperature (PF). (*n* = 3 ± STD).

Moisture loss is known to be attributable to the firming of bread during the storage period. As previously mentioned, firming is mainly caused by the retrogradation of amylopectin and moisture redistribution or loss and describes the increasing hardness of the crumb with increasing storage time [2]. During baking, starch gelatinization occurs, which refers to the process of the melting of native amylopectin crystals. Therefore, amylopectin is in an amorphous plasticized conformation after baking. Reversing this, recrystallization occurs during bread storage. The side chains of the amylopectin molecules reorganize, which results in the formation of double helices of the outer branches and increases the rigidity. Next to this, an increased formation of B-type crystalline polymorphs can be observed due to the retrogradation process. This crystal structure is capable of immobilizing more water molecules than A-type crystals [5,34] and reinforces the adverse effect of a firmer crumb texture. One measure for the hardness of the crumb is firmness and this can be accessed by texture profile analysis. As presented in Figure 1, an overall trend can be seen in which the crumb firmness increased during the storage period of 10 days for all storage conditions and bread types.

The comparison of the storage methods indicates an increased firming rate for storage at fridge temperature (Figure 1). The firmness for mixed-type sourdough bread, stored in a plastic bag at fridge temperature, increased to 398% and for pan wheat bread to 424%. It is commonly known that low temperatures accelerate the firming rate in starch gels [35]. Gray and Bemiller stated that for starch gels in bread-like concentrations, retrogradation occurs at temperatures above the glass transition temperature (Tg, −5 °C) and below the melting temperature (Tm, 60 °C) [20]. The recrystallization process during starch retrogradation involves nucleation and propagation processes, the rates of which are reported to be highest for temperatures close to Tg and Tm, respectively. Slade and Levine (1987) concluded from their experiments that the starch retrogradation rate, as a combination of nucleation and propagation processes, is higher for refrigerator temperatures and, therefore, the storage of bread at refrigerator temperatures accelerates firming [34]. As expected, the storage at room temperature resulted in considerably lower firming rates. The second highest firming rate was found for storage in PE-layered microperforated paper bags. As this storage method caused the highest moisture loss, as can be seen from Table 2, it is evident that the drying out contributed to an accelerated firming of the crumb. Overall, the lowest firming rates were found for storage in a bread box and in a sealed plastic bag. In general, starch retrogradation bag under these two storage conditions is assumed to proceed at the same rate as for bread stored in a layered paper bag, but drying of the crumb was limited due to the hindered air circulation. This was also indicated by the higher humidity inside these storage containers. In total, the lowest firming rate was observed for the plastic bag at room temperature. For this method, the firmness of the mixed-type sourdough bread increased to 330% compared to the initial firmness and the pan wheat bread increased to 390%. For all storage methods, the firming behavior of pan wheat bread, especially when stored in a bread box at room temperature, was found to differ from the linear model (storage time ≥ 5 days). This could have been caused by the occurrence of degradation processes upon the growth of molds and yeasts.

**Figure 2:**
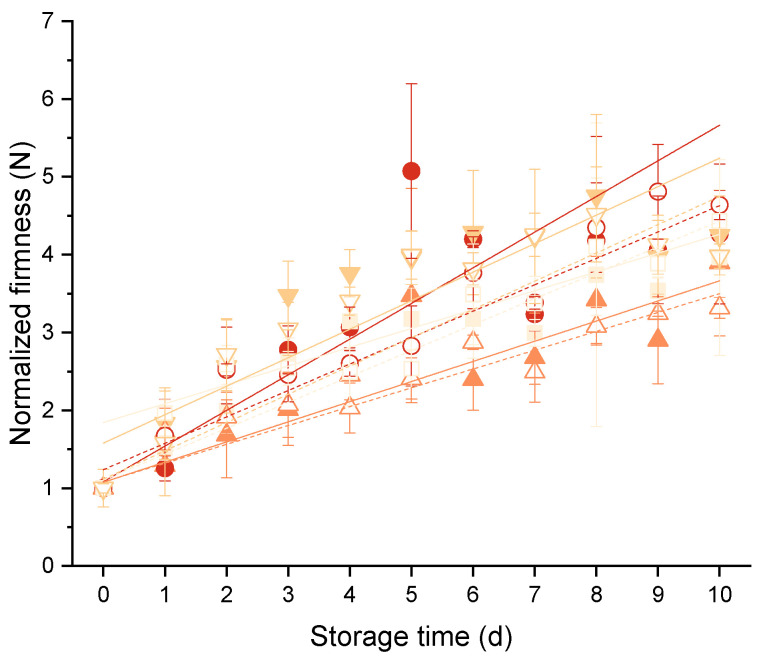
Normalized bread firmness as a function of the storage time depending on the storage conditions applied. Filled symbols and solid lines represent pan wheat bread samples, open symbols, connected by dashed lines, represent mixed-type sourdough bread samples. (■) Bread box at room temperature (BB), (●) PE-layered microperforated paper bag at room temperature (LP), (▲) plastic bag at room temperature (PR) and (▼) plastic bag fridge temperature (PF). Normalized firmness of the crumb over storage time was fitted using a linear model.

Overall, the absolute firming rates were markedly higher for mixed-type sourdough breads (cf. Table 3). This observation can be ascribed to the differences in crumb structure within wheat and mixed-type breads. The denser crumb structure of the mixed -type sourdough breads can be translated into a higher material/volume unit ratio. Jekle et al. (2018) suggested that this causes an apparently higher firming rate due to methodical influence [36]. As suggested by these authors, normalization can be used as tool to correct textural measures for structural influences. The results of the normalization are presented in Figure 2 and Table 4. After the structural correction, the normalized firming rates of mixed-type sourdough breads were even found to be slightly lower than the normalized firming rates of wheat bread. This might have been caused by differences in major crystallites between rye and wheat breads. Further, the relative crystallinity of starch in rye sourdough bread is less and was found to increase more slowly than in wheat bread [37]. Additionally, pentosans have been reported to reduce the firmness of supplemented breads, even though the higher moisture content of these systems was not corrected in these studies. This might have influenced the outcomes of this study, as pentosanes bind more water and therefore reduce the mobility of starch in the bread crumb matrix. This can probably contribute to the decelerated recrystallization process [38]. Nevertheless, the use of mixed-type sourdough breads might have caused only minor differences in the firming behavior.

The excesses of retrogradation and drying out were both found to vary for the different storage methods. The results indicate that the prevention of the drying of the crumb by suitable barrier materials can retard the firming process. Further, the reduction of the storage temperature to fridge temperature was shown to accelerate the retrogradation process. The faster recrystallization clearly impacted the firmness of the samples and accelerated the firming process.

## 4. Conclusions

Bread is a staple food consumed worldwide and has recently been reported to contribute to the increasing amount of food waste. This can be attributed to staling and spoilage processes that take place during the storage of bread. The objective of this study was to investigate the influence of storage parameters on staling and spoilage behavior of bread within the limits of common household methods. The design of this study aimed at providing an objective comparison of household storage methods with opposable data to address superficial knowledge and common prejudges. Visual inspection was shown to represent an adequate tool for microbial safety control. Even though fungal growth was quantifiable before it was perceived visually, no health risk was related to this as molds do not represent a health risk on their own. Instead, mycotoxins are commonly associated with adverse health outcomes, resulting in, e.g., carcinogenic or mutagenic effects. The production of mycotoxins is not directly linked to the growth of fungi. Therefore, the microbial safety of food is hard to access for consumers and misjudgment or excessive caution can cause preventable food waste. No mycotoxins were quantified in bread slices after ten days of storage in plastic bags at room temperature, even though this storage method resulted in excessive spoilage. It can be concluded that disgust can prevent any foodborne health risk, as visual appearance can prompt the avoidance of the consumption of spoiled food before mycotoxins become detectable at quantifiable concentrations. The obtained data suggest, therefore, that precautionary disposal of stored bread without any visually accessible mold growth indication is not necessary. Further, we have shown that it is possible to increase the shelf life of bread products within the limits of common household storage methods. The fact that the proper temperatures for reducing fungal growth accelerate the firming processes limits the possibilities for appropriate storing methods. Thus, the presented results rule out some possibilities for prolonging shelf life. It has been demonstrated that humid atmospheres promote spoilage. Therefore, appropriated construction of bread boxes or packaging materials (e.g., improved water permeability or moisture scavengers) can help to extent the shelf life of bread. In general, the correct choice of bread type for long storage times contributes to the reduction of food waste and sourdough and rye specifically have been shown to decelerate spoilage and staling processes. Further, sanitary conditions are essential for the storage of bread and represent a simply accessible way to prolong the stability of bread. The presented results may eventually lead to a more prudent use of storage methods.

## Figures and Tables

**Table 1:** Results of initial bread characterization ($\bar{x}$ ± STD, *n* = 3).

	Moisture Content (%)	a_w_-Value ( )	pH-Value ( )	Total Titratable Acidity (mL 0.1 M NaOH)
Pan wheat bread	45.38 ± 2.60 ^a^	0.98 ± 0.01 ^a^	5.48 ± 0.03 ^a^	4.18 ± 0.31 ^a^
Mixed-type sourdough bread	48.59 ± 0.01 ^a^	0.97 ± 0.01 ^b^	4.48 ± 0.06 ^b^	9.11 ± 0.28 ^b^

^a,b^ Mean values ± STD labelled with a different letter in the same column are significantly different according to Tuckey’s test on a significance level of α = 0.05.

**Table 2:** Absolute moisture loss of bread samples (crumb and crust) for different storage conditions and bread types after a storage period of ten days ($\bar{x}$ ± STD, *n* = 3).

	Absolute Moisture Loss of Pan Wheat Bread (%)	Absolute Moisture Loss of Mixed-Type Sourdough Bread (%)
PE-layered microperforated paper bag (21.3 ± 0.4 °C)	10.3 ± 2.9	13.5 ± 3.3
Bread box (22.0 ± 0.5 °C)	7.0 ± 2.9	8.9 ± 1.3
Plastic bag (21.9 ± 0.5 °C)	3.5 ± 3.1	5.7 ± 0.6
Plastic bag (8.2 ± 0.2 °C)	3.1 ± 2.5	5.5 ± 1.1

**Table 3:** Absolute firming rates for different storage conditions and bread types. The results are shown in the table in terms of the firming rate (N/d, increase in firmness per day of storage), defined as the slope of the linear model ($\bar{x}$ ± STD, *n* = 3).

	Absolute Firming Rate of Pan Wheat Bread (N/d)		Absolute Firming Rate of Mixed-Type Sourdough Bread (N/d)	
PE-layered microperforated paper bag (21.3 ± 0.4 °C)	0.51 ± 0.07	(R² = 0.84)	1.30 ± 0.14	(R² = 0.90)
Bread box (22.0 ± 0.5 °C)	0.27 ± 0.07	(R² = 0.61)	1.27 ± 0.12	(R² = 0.93)
Plastic bag (21.9 ± 0.5 °C)	0.29 ± 0.03	(R² = 0.92)	0.92 ± 0.05	(R² = 0.98)
Plastic bag (8.2 ± 0.2 °C)	0.41 ± 0.08	(R² = 0.76)	1.38 ± 0.16	(R² = 0.89)

**Table 4:** Firming rates for different storage conditions and bread types after normalization. The results are shown in the table in terms of the firming rate, defined as the slope of the linear model ($\bar{x}$ ± STD, *n* = 3).

	Norm. Firming Rate of Pan Wheat Bread (N/d)		Norm. Firming Rate of Mixed-Type Sourdough Bread (N/d)	
PE-layered microperforated paper bag (21.3 ± 0.4 °C)	0.46 ± 0.07	(R² = 0.84)	0.34 ± 0.04	(R² = 0.90)
Bread box (22.0 ± 0.5 °C)	0.24 ± 0.06	(R² = 0.61)	0.33 ± 0.03	(R² = 0.93)
Plastic bag (21.9 ± 0.5 °C)	0.26 ± 0.03	(R² = 0.92)	0.24 ± 0.01	(R² = 0.98)
Plastic bag (8.2 ± 0.2 °C)	0.37 ± 0.07	(R² = 0.76)	0.36 ± 0.04	(R² = 0.89)

## Data Availability

Data is contained within the article.
